# A Rare Cheek Sinus Presentation of a Type II First Branchial Cleft Anomaly: A Case Report and Literature Review

**DOI:** 10.3390/jcm14238519

**Published:** 2025-12-01

**Authors:** Mor Rittblat, Bar Davidov, Menahem Gross, Noam Armon

**Affiliations:** 1 Department of Plastic and Reconstructive Surgery, Hadassah Hebrew University Medical Centre, Jerusalem 91120, Israel; bard@hadassah.org.il (B.D.); rnoama@hadassah.org.il (N.A.); 2 Department of Military Medicine and “Tzameret,” Faculty of Medicine, The Hebrew University of Jerusalem, Jerusalem 91120, Israel; 3 Department of Otolaryngology Head and Neck Surgery, Hadassah-Hebrew University Medical Center, Jerusalem 91120, Israel; gross@hadassah.org.il; 4 School of Medicine, Hebrew University, Jerusalem 91120, Israel

**Keywords:** congenital cheek sinus, branchial cleft anomaly, accessory tragus, pediatric plastic surgery

## Abstract

**Background:** Congenital cheek sinuses are exceedingly rare craniofacial anomalies, typically present at birth and often misdiagnosed due to their resemblance to dermal pits or dimples. Only a few cases have been reported in the literature. This case is notable for its histopathological confirmation as a type II first branchial cleft anomaly containing an accessory tragus, highlighting the embryologic and clinical overlap between congenital cheek sinuses and branchial cleft anomalies. **Case presentation:** We report a three-year-old boy presenting with a congenital dimple along the right nasolabial fold. The lesion was present since birth and occasionally discharged clear fluid but had no history of infection or inflammation. Imaging demonstrated a subcutaneous tract without communication to the oral cavity or parotid gland. Under general anesthesia, surgical excision was performed, and the sinus tract was dissected in continuity to a cartilaginous remnant. Histopathologic analysis confirmed the presence of an accessory tragus consistent with a type II first branchial cleft anomaly. The patient’s postoperative recovery was uneventful, and at three months’ follow-up, there was no recurrence and an excellent cosmetic outcome. **Conclusions:** This case emphasizes the importance of considering branchial cleft anomalies in the differential diagnosis of congenital cheek sinuses. Although these lesions may appear benign and superficial, histopathological confirmation is critical for accurate classification. Complete surgical excision remains the definitive treatment, ensuring both diagnostic clarity and favorable aesthetic results. Increased awareness of such rare anomalies can improve diagnostic accuracy and surgical planning, particularly with respect to their potential proximity to critical facial structures.

## 1. Background

Congenital sinuses of the face are uncommon developmental anomalies, most frequently observed in the midline or paramedian regions of the upper or lower lip [1]. In contrast, congenital cheek sinuses are rarely encountered and sparsely reported in the literature with precise incidence remains undefined [2,3,4]. These lesions may occur independently or as part of a broader syndrome involving craniofacial development, such as oculo-auriculo-vertebral spectrum [5] also known as the Goldenhar-Gorlin syndrome [5,6] or branchio-oto-renal syndrome [7].

The embryologic origin of facial fistulas and sinuses and related anomalies such as branchial cleft anomalies is attributed to aberrant or incomplete fusion of the maxillary and mandibular prominences, which arise from the first and second branchial arches during early embryogenesis [3,8]. Some authors have proposed that congenital cheek fistulas/sinuses may also result from persistent ectodermal-lined tracts associated with failed regression of tissues near the parotid or accessory salivary system [2,8,9,10].

Clinically, these lesions may present with intermittent or continuous serous or mucopurulent discharge, particularly when secondarily infected [2,3,4]. In asymptomatic cases, cosmetic concerns are often the primary indication for evaluation. The differential diagnosis includes dermoid cysts, preauricular sinuses, salivary sinuses, and first branchial cleft anomalies [4].

First branchial cleft anomalies are rare congenital malformations of the first pharyngeal cleft, accounting for fewer than 10% of all branchial cleft lesions [11,12,13]. Work classified first branchial cleft anomalies into two types according to their embryologic origin, histologic features, and relationship to the external auditory canal [11]. Type I lesions are purely ectodermal duplications of the membranous external auditory canal and usually present as cystic periauricular masses lined by squamous epithelium without skin adnexa or cartilage [11,12,14]. Type II lesions contain both ectodermal and mesodermal components and typically present as cysts, sinuses, or fistulae in the periauricular, parotid, or submandibular region, often in close relation to the facial nerve and external auditory canal [11,12,14].

Imaging modalities such as ultrasound or MRI can help define the tract’s extent and exclude communication with deeper structures such as the buccal mucosa or parotid gland [13,15].

Herein, we report a case of a three-year-old male with a congenital cheek lesion initially suspected to be a lateral facial sinus. Surgical excision and subsequent histopathologic analysis identified the lesion as a type II first branchial cleft anomaly. This case underscores the embryologic and clinical overlap between congenital cheek sinuses and branchial cleft anomalies and highlights the importance of histopathological confirmation in guiding accurate diagnosis and management.

## 2. Case Presentation

**Figure 1:**
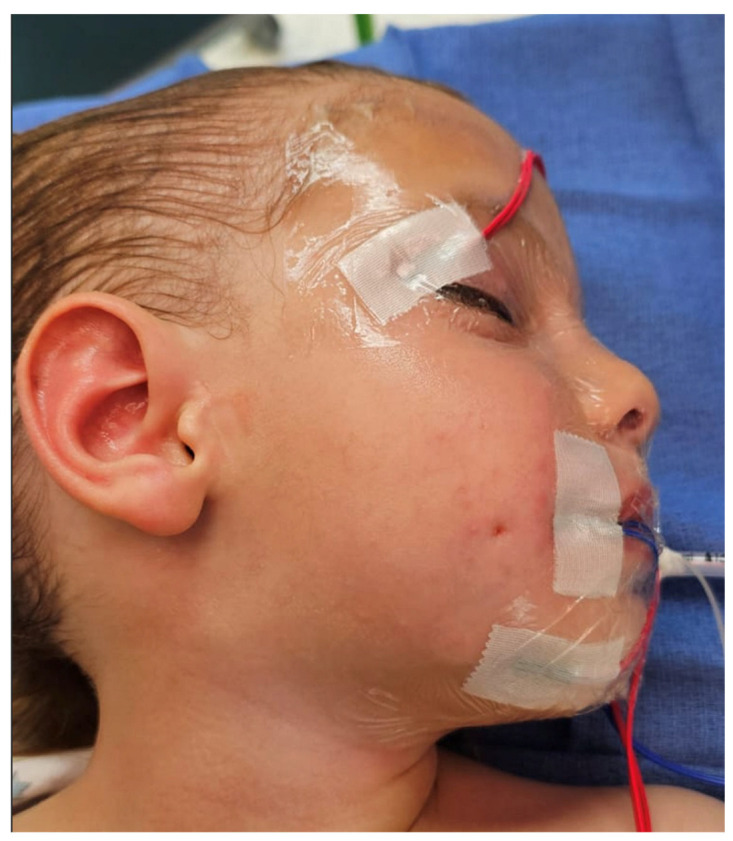
Preoperative photograph illustrating the congenital cheek sinus.

A three-year-old male was referred to our pediatric plastic surgery clinic for evaluation of a congenital dimple on the right cheek (Figure 1).

The patient was born with dysmorphic features initially raising suspicion for Goldenhar syndrome. However, after multidisciplinary evaluation, including genetic consultation and imaging evaluation, Goldenhar syndrome was ruled out. Growth and developmental milestones were reported to be appropriate for age. On examination, facial symmetry was preserved, and oral motor function was normal. A small cutaneous punctum was observed along the nasolabial fold, without evidence of inflammation. The lesion, present since birth, was located approximately 2 cm lateral to the oral commissure and measured about 2 mm in diameter. According to the parents, it occasionally discharged clear fluid, but there was no history of swelling, erythema, pain, or infection. Ultrasound imaging revealed a subcutaneous tract extending approximately 1.5 cm in depth. There was no communication identified with the buccal mucosa, parotid gland, or oral cavity, and no associated cystic lesion was seen.

### 2.1. Surgical Technique

Under general anesthesia, without neuromuscular blockade, the patient was positioned supine, and facial nerve monitoring was applied. Following aseptic preparation and draping, the skin was incised according to preoperative markings.

**Figure 2:**
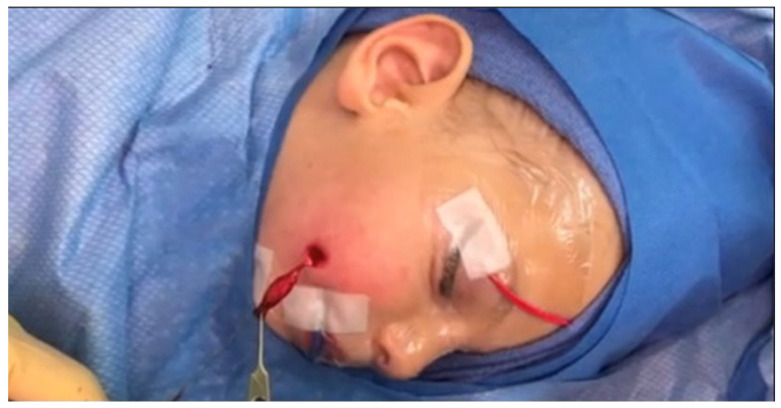
Intraoperative photograph during excision of the congenital cheek sinus.

The punctum was identified at the lower and slightly lateral border of the right nasolabial fold, with no discharge or signs of infection. A lacrimal probe was inserted into the punctum, delineating a sinus tract measuring approximately 3 cm, oriented toward the parotid region. Gentle dissection was performed along the tract, progressing laterally and deep to the zygomaticus major and buccinator muscles. The tract terminated in a cartilaginous remnant and was excised in its entirety without breaching its lumen (Figure 2, Video S1). Hemostasis was achieved using bipolar cautery.

Following irrigation and debridement, layered closure was performed: subcutaneous tissue was approximated with 4-0 Monocryl sutures, and the skin with 6-0 nylon. A sterile adhesive dressing was applied. The patient remained hemodynamically and respiratory stable throughout the procedure, and facial nerve function was preserved. The patient was transferred in stable condition to the recovery unit.

**Figure 3:**
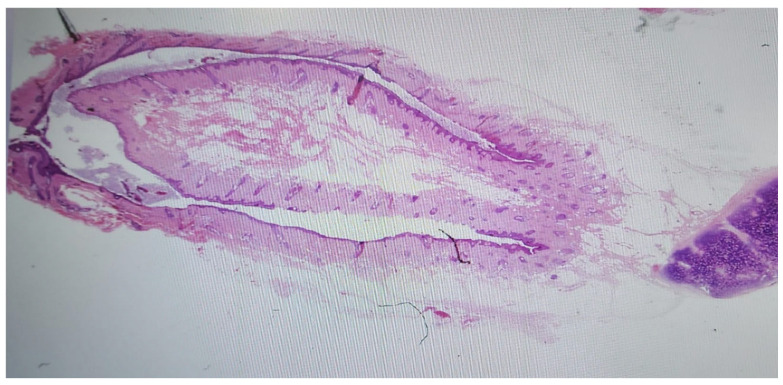
Histologic photograph of the excised specimen.

Histopathologic examination revealed a 2.4 × 0.8 × 0.6 cm cylindrical sinus tract, consistent with an accessory tragus, representing a type II first branchial cleft anomaly (Figure 3).

### 2.2. Outcome

**Figure 4:**
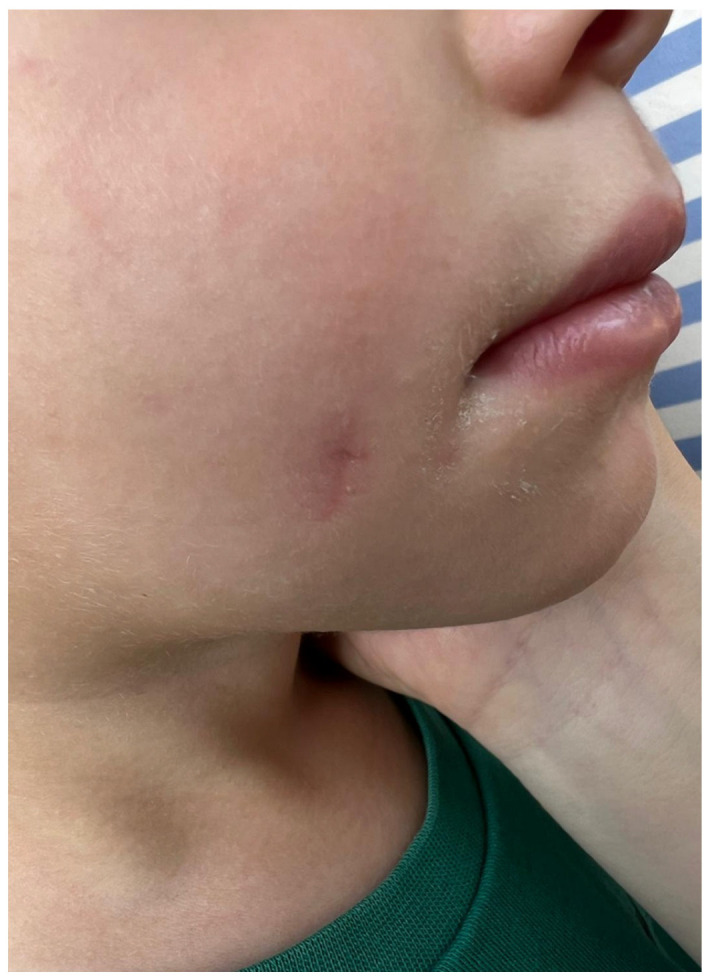
Postoperative photograph at three months follow-up.

The postoperative course was uneventful. The patient was followed up at regular intervals, and at three-month follow-up, no signs of recurrence were observed. The cosmetic result was excellent, with no noticeable deformity of the cheek (Figure 4).

## 3. Discussion

Type II first branchial cleft anomalies are rare congenital lesions resulting from incomplete obliteration of the cleft between the first and second branchial arches during embryogenesis [8,11,12]. These anomalies often present near the external auditory canal or parotid gland but may occasionally appear in atypical locations along the line of embryologic fusion, including the cheek [4,10,13]. Their clinical presentation can mimic other congenital lesions such as dermoid cysts, salivary fistulas, or ectopic accessory tragi [14]. Embryologically, type II anomalies are thought to arise from persistence of both ectodermal and mesodermal elements of the first branchial cleft. They frequently contain keratinized squamous epithelium, skin adnexal structures, and cartilage, and may course close to the facial nerve or parotid gland [4,8]. When located outside the typical periauricular region, such as in the center of the cheek or along the nasolabial fold, these anomalies may be misdiagnosed as simple sinus tracts or dermal pits.

Unlike accessory tragi, which are usually superficial, isolated lesions without deep extensions or communication with adjacent structures, type II branchial cleft anomalies may track through deeper tissue planes and have a more complex anatomical course. In our case, although the lesion was superficial and had a simple appearance, histopathological analysis revealed an accessory tragus inside a sinus, features consistent with an extremely rare type II first branchial cleft anomaly [4]. Review of the limited literature reveals that cheek presentations of branchial cleft anomalies are exceedingly rare and often underrecognized due to their variable location and overlap with other congenital lesions. While some may be associated with syndromic features such as branchio-oto-renal syndrome [7] or Goldenhar syndrome [5,6]. In this case, syndromic involvement was ruled out, and the patient exhibited normal development and anatomy aside from the facial lesion. However, the possibility of an underlying genetic syndrome cannot be entirely excluded and may emerge in the future, underscoring the importance of continued research and maintaining a high index of suspicion.

Diagnosis is primarily clinical, with imaging reserved for deeper or ambiguous lesions to evaluate extent and rule out communication with critical structures. Histopathologic examination remains essential for definitive classification. Surgical excision is the treatment of choice and is typically curative, offering both diagnostic clarity and cosmetic improvement.

This case underscores the importance of including first branchial cleft anomalies in the differential diagnosis of congenital facial sinuses or dimples, particularly when located along embryologic fusion planes of the branchial apparatus. Accurate classification is essential for surgical planning, especially given the potential proximity to the facial nerve.

## 4. Conclusions

We report a rare case of an ectopic accessory tragus located in a cheek sinus. While accessory tragi are typically found in the preauricular area, atypical locations along the migration pathway of the first branchial arch can result in diagnostic confusion. This case highlights the clinical and embryological overlap between accessory tragi and other congenital cheek anomalies. Accurate diagnosis relies on histopathological confirmation, and surgical excision remains the definitive treatment, providing both diagnostic clarity and excellent cosmetic outcomes. Clinicians should consider accessory tragus in the differential diagnosis of congenital facial dimples tracts or sinuses, especially when located along embryological fusion lines.

## Data Availability

Full demographic data pertaining to individuals cannot be disclosed in order to ensure subjects’ anonymity and data security policies. Derived data supporting the findings of this study may be made available from the corresponding author MR upon request.

## Supplementary Materials

The following supporting information can be downloaded at: https://www.mdpi.com/article/10.3390/jcm14238519/s1, Video S1: Intraoperative video demonstrating excision of the fistula.
